# Impacts of COVID-19 restrictions on level of physical activity and health in home-dwelling older adults in Norway

**DOI:** 10.1186/s11556-022-00309-w

**Published:** 2022-12-09

**Authors:** Arnhild J. Nygård, Kristin Taraldsen, Randi Granbo, Geir Selbæk, Jorunn L. Helbostad

**Affiliations:** 1 Department of Neuromedicine and Movement Science, Faculty of Medicine and Health Sciences, Norwegian University of Science and Technology (NTNU), Trondheim, Norway; 2 Department of Physiotherapy, Oslo Metropolitan University (OsloMet), Oslo, Norway; 3 Department of Geriatric Medicine, Oslo University Hospital, Oslo, Norway; 4 Norwegian National Centre for Ageing and Health, Vestfold Hospital Trust, Tønsberg, Norway; 5 Faculty of Medicine, University of Oslo, Oslo, Norway

**Keywords:** Older adults, Physical activity, Health, COVID-19

## Abstract

**Background:**

The spread of the coronavirus in spring 2020 led to a lockdown of physical activity (PA) offers. The aim of this study was to investigate how PA, as well as general and mental health, in community-dwelling older adults were affected by the COVID-19 restrictions in Norway.

**Methods:**

Invitation to participate in the study was sent via Facebook and the Norwegian Pensioners’ Association. Inclusion criteria were being ≥ 65 years old and living at home. Participants completed a questionnaire either digitally or on paper in June–August 2020. The questionnaire included questions on PA, general health, and mental health both before (13th of March) and during lockdown.

**Results:**

We included 565 participants (mean age 74 ± 5.3 years, 60.4% female); almost 60% had a university degree, 84% reported performing PA more than once per week, and 20% reported a fall in the previous 12 months. The Wilcoxon signed-rank test indicated that the corona lockdown significantly reduced activity level (Z = -4.918, *p* < 0.001), general health (Z = -6,910, *p* < 0.001) and mental health (Z = -12.114, *p* < 0.001). Those who were less active during lockdown had higher odds of experiencing worse health than those who maintained their activity level, odds ratio: 9.36 (95% CI = 4.71–18.58, *p* < 0.001) for general health and 2.41 (95% CI = 1.52–3.83, *p* < 0.001) for mental health. Those who attended organized exercise offers before lockdown had higher odds of being less active during lockdown compared to those who did not exercise in an organized setting, odds ratio: 3.21 (95% CI = 2.17–5.76, *p* < 0.001).

**Conclusions:**

In a relatively highly educated and active group of older participants, COVID-19 restrictions still negatively affected level of activity as well as general and mental health. These short-term decreases in activity level and health suggest that preventive actions and increased focus on measures to support older adults in maintaining an active lifestyle are needed.

## Background

Physical activity (PA) and exercise are effective measures for maintaining and improving general health status and for preventing a wide range of diseases [1]. Caspersen and colleagues’ define PA as any bodily movement produced by skeletal muscles that results in energy expenditure, and could be f.ex. gardening, walking or doing sports, while exercise is defined as a subset of PA that is planned, structured and repetitive, and done to improve or maintain physical fitness [2]. PA and exercise are important contributors to maintaining physical function in older age, which is essential for independence in activities of daily living (ADL) and preventing falls [3, 4]. In addition, evidence strongly indicates that PA improves health-related quality of life in old age [5]. Still, only 32% of Norwegian older adults meet the national recommendations of engaging in 150 min of PA per week [6].

Older adults are among those who are most vulnerable to experiencing a rapid decline in physical function due to sedentariness or low activity levels; this decline can, in turn, cause a loss of independence and an increased number of falls [7]. Systematic reviews have shown that older adults who are physically active have reduced risks of disability related to ADL, functional limitations, and recurrent falls; these reviews have also shown that higher PA and lower sedentary behavior are associated with greater ability to complete ADL [7, 8]. Morey and colleagues found that participants who went from more than 150 min of PA per week to less than 150 min per week over the course of 6 months experienced a 11.8-point drop in health-related quality of life, as measured by the SF-36 [9].

In December 2019, the world received news about a new coronavirus (COVID-19) that originated in Wuhan, China. In 2020, the year began with a steady spread of the virus to other countries, including Norway. In March 2020, COVID-19 was classified as a global pandemic by the World Health Organization (WHO), and on March 13, Norway implemented the strictest peacetime measures to prevent a massive spread of the virus. This lockdown included recommendations on social distancing, closed gyms, and a temporary halt in all organized offers of PA and exercise, among other things. Here we use Collins Dictionary’s definition of organized activity; it involves a number of people doing something together in a structured way, rather than doing it by themselves. This could be exercise groups, walking groups or exercising at the gym [10]. People with high risk of becoming seriously ill from COVID-19, including those aged 65 years and older, were recommended to self-isolate [11]. The Norwegian government still advised people to continue doing PA or exercise in their homes or outside as long as social distancing were possible.

Lockdowns and social distancing are effective ways of slowing the spread of the virus [12]. However, this method may have secondary effects on other dimensions of people’s health. Being confined to one’s home and losing all organized offers of PA and exercise could have consequences for both the general population’s and older adults’ level of PA, which has been found in several countries during the COVID-19 pandemic [13, 14]. As global trends show that many does not meet the guidelines for PA we might and up with “a tale of two pandemics” with the added COVID-19 restrictions, as Hall and colleagues address it [15, 16]. The COVID-19 situation presents a natural experiment that provides an opportunity to investigate how a limited amount of PA and exercise offers affects levels of activity and health. Evidence is lacking on how environmental factors—such as restrictions in society—affect how active older adults are and how such restrictions affect health in older adults living independently. Thus, the aim of this study was to investigate how physical activity, general health, and mental health in community-dwelling older adults were affected by the lockdown during the COVID-19 pandemic in Norway. Furthermore, we wanted to investigate how maintaining one’s activity level during lockdown affected their general and mental health status. Lastly, because people were allowed, and advised to, continue doing exercise in their homes or outside at the same time as all organized offers were temporarily shut down, we wanted to investigate if the type of PA the older adults engaged in before lockdown—i.e., organized offers or non-organized offers—affected their activity levels during lockdown.

## Method

### Design and procedure

This study is a survey with the inclusion criteria being 65 years or older and living at home in Norway. The study was approved by the Central Regional Committee for Medical and Health Research Ethics (REC Central—10418/REK midt). Participants were given written information about the project and gave an active consent by checking of a box (“I have read the information and consent to participate”) before they obtained access to the questionnaire. If a participant checked the box “I do not wish to participate”, no other questions were available, and the questionnaire was ended.

Participants completed a questionnaire either digitally or on paper in the period from June to August 2020. Recruitment to the digital questionnaire was conducted through snowball sampling, using social media and newsletters. A link to the digital questionnaire was distributed via various Facebook pages: the Norwegian Pensioners’ Association (NPA), “Sterk og stødig” (Strong and Steady, a national preventive group exercise concept for older people), and the author’s personal pages. In addition, the NPA included information on the study and the link to the survey in their monthly newsletter. The paper version of the questionnaire was distributed and collected through the NPA’s local offices in three counties (Møre og Romsdal, Agder, and former Østfold in Viken). All paper versions were kept in sealed envelopes during transport and then stored in a locked cabined in a locked room. The NPA hired someone (who were registered as a project employee at REC Central) to manually plot the data from the paper questionnaire to a digital format.

The digital questionnaire was created using www.nettskjema.no, a survey solution developed and hosted by the University of Oslo (nettskjema@usit.uio.no). The data were stored on the TSD (Tjeneste for Sensitive Data) facilities, owned by the University of Oslo and operated and developed by the TSD service group at the University of Oslo’s IT Department (tsd-drift@usit.uio.no).

### Measures

The questionnaire included items on general health, mental health, and physical activity. Items on background, the HUNT 1 Physical Activity Questionnaire (HUNT 1PA-Q) [17] and Cohort NORway Mental Health Index (CONOR-MHI) [18] were taken from the HUNT survey, a large population-based longitudinal study involving 240,000 participants since 1984 [19]. (The HUNT questionnaires can be found at https://www.ntnu.edu/hunt/data/que.) Most questions were asked for both before the 13^th^ of March and after/present-day at the time of the survey. The questionnaire used in the current study consisted of three parts.

Part One included 13 questions on background information, such as age, gender, living conditions, the use of walking aids, falls in the previous 12 months (0/1/2/3/ > 3), fear of falling (not at all/yes a little/yes pretty worried/yes quite worried), changes in cognition in the last five years (none/mild/moderate/severe), and ADL. ADL was assessed by the mobility subscale plus three items from the domestic subscale of the Nottingham Extended ADL Index (Nottingham EADL) [20].

Part Two included 10 items on PA and exercise, including the amount of PA and exercise in general along with the amount of different specific activities (e.g., walking, biking, gym, and group exercise). The amount of PA was measured with the HUNT 1 PA-Q which includes questions on frequency (0.0–5.0 points), intensity (1–3 points) and duration (0.10–1.00 points) of PA. These three subscales together gives an index with a score ranging from 1.1–15, with 15 as the best score [17]. (Example item: “Before the 13^th^ of March, how often did you do physical exercise?”).

Part Three included six items on general and mental health. (Example item: “Today, most of the time, how would you say your health is?”) For general health, we used the first question from the SF-36 [21], and for mental health, we used the seven questions from the CONOR-MHI. Each of the CONOR-MHI items are rated on a Likert Scale (1–4), with total scores ranging from 7–28, where higher scores indicate worse mental health [18].

The questionnaire also included items on what motivates participants to perform physical activity and exercise, but this was not the focus of this paper, thus these items are not reported here.

### Data processing and analysis

Descriptive analyses include participants’ demographic characteristics, presented as numbers and percentages for the total sample and by gender. Some categories were merged because of few answers or practical means; for education “high school” and “apprenticeship”, for living conditions “with husband” and “with others”, for number of falls 2, 3 and > 3 falls, for fear of falling “pretty worried” and “quite worried” and for change in cognition “moderate change” and “severe change” were merged. The Chi-square test was used to examine differences in background variables for gender.

We analyzed changes in PA, general health, and mental health before versus after the lockdown using the Wilcoxon Signed-Rank Test. Logistic regression was used to analyze how maintaining one’s activity level affected their general and mental health status during lockdown, as well as how the type of activity before lockdown (organized offer exercise or non-organized exercise) affected activity levels during lockdown. Independent variables with a bivariate Spearman’s correlation of > 0.1 with the dependent variable were included as covariates in the logistic regression analyses: these were gender, general health, and type of activity before lockdown.

For the regression analyzes, based on the participants’ answers on HUNT 1 PA-Q from before 13^th^ of March and today a new variable was created and labeled as change in PA. From this new variable the participants were divided into two groups; those who reported a reduced levels of PA, and those who reported the same or increased levels of PA. Based on the participants’ answers related to general health and CONOR-MHI from pre-lockdown and at the time of the questionnaire, two new variables were created and labeled as change in general health and change in mental health. For these two new variables, the participants were divided into two groups: those who reported having worse general or mental health currently compared to pre-lockdown, and those who reported the same or better general and mental health currently compared to pre-lockdown.

We also divided the participants based on how frequently they attended organized offers of exercise before the lockdown. The two new groups were labeled as “organized offer exercisers” (those attending the gym or group exercise classes once or more per week) and “non-organized exercisers” (those attending the gym or group exercise classes less than once a week).

All statistical analyses were computed using IMB SPSS 27 (IBM Corp. Released 2020. Armonk, NY: IBM Corp), and the significance level was set at *p* < 0.05.We did not perform any power calculation as we included participants within a limited timeframe, to ensure similar conditions across participants regarding restrictions.

## Results

Of the 585 respondents, 20 were excluded due to the age criteria: 12 were younger than 65 years, and eight did not report their age. This left us with 565 participants for the analysis. Most participants (86%) completed the digital version of the questionnaire, and most participants heard of the study through NPA (61.6%) and social media (19,6%).

Table 1 shows the demographics of the total sample (*n* = 565) and the sample split by gender. Participants’ mean age was 74 (± 5.3) years (range: 65–95 years), and 60.4% were women. Almost 60% had a university education, and 84% reported being physically active more than once per week before the lockdown. The participants were largely independent in ADL, with more than 90% answering that they performed the selected activities from the Nottingham EADL alone without any problems. One exception was ‘using public transport’, where 74% reported that they did this independently. Less than 10% used a walking aid, and about 20% had experienced a fall in the previous 12 months. About 50% were “not at all afraid of falling”.

**Table 1:** Demographic data

	Total sample (*n* = 565)		Men (*n* = 220)		Women (*n* = 341)	
**Age (Mean, SD)**	73.64 ± 5.29	Range 65–95	74.36 ± 5.15	Range 65–90	73.17 ± 5.36	Range 65–95
	*n*	%	*n*	%	*n*	%
**Education**						
Primary school	37	6.5	14	6.4	23	6.7
High school/Apprenticeship	195	34.5	75	34.1	120	35.2
University	329	58.2	128	58.2	197	57.8
**Living**^**a**^						
Alone	214	37.9	50^a^	44,764	164	48.1
With husband or others	348	61.5	168	75.9	175	50.8
**Use of walking aid**						
None	515	91.2	204	92.7	307	90.0
Yes, outdoor	49	8.7	15	6.8	34	10.0
Yes, indoor	11	1.9	2	0.9	9	2.6
Number of falls*						
0	447	79.1	184	83.6	259	76.6
1	*54*	9.6	*16*	7.3	*38*	11.1
2 or more	60	10.6	19	8.6	41	12.0
**Fear of falling**^**a**^						
No, not at all	317	56.1	154	70.0	160	46.9
Yes, a little	215	38.1	61	44,769	153	44.9
Yes, pretty/quite	33	5.8	3	1.4	19	5.6
**Change in cognition last 5 yrs**^**b**^						
None	215	38.2	75	34.1	139	40.8
Mild	283	49.8	106	48.2	175	51.6
Moderate/severe	65	11.5	39	17.7	25	7.4

^a^Chi-square difference in gender *p* < 0.001

^b^Chi-square difference in gender *p* = 0.001

^*^Chi-square difference in gender *p* = 0.045

More women than men lived alone, reported a fear of falling, and had experienced one or more falls the previous 12 months. More men than women reported a change in cognition in the last five years.

### Changes in levels of physical activity and health

**Fig. 1:**
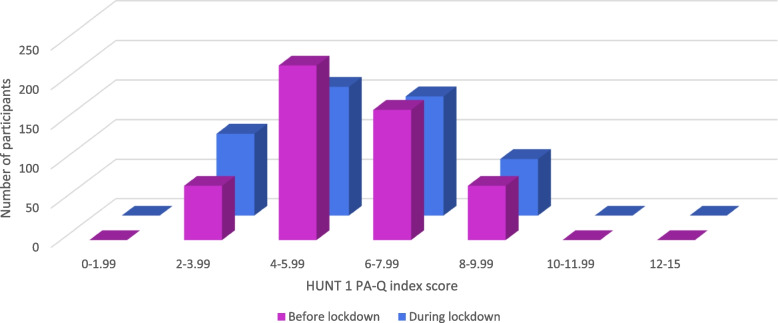
PA before and during the lockdown. Distribution of participants’ HUNT 1 PA-Q index scores before and during lockdown (1.1–15), where 15 is the best score. No participants had lower than 2 or higher than 9 points

**Fig. 2:**
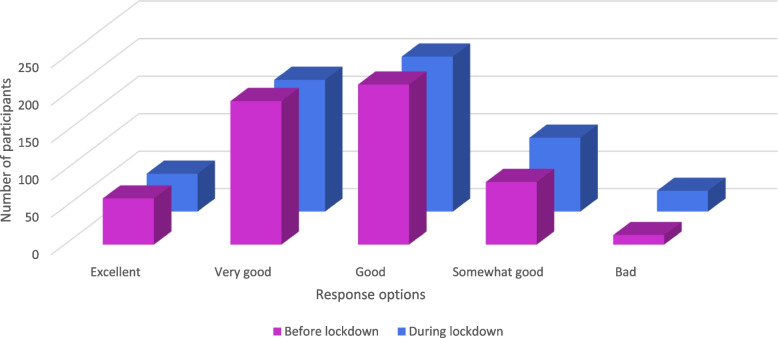
General health before and during the lockdown. Distribution of participants’ answers on SF-36’s first item

**Fig. 3:**
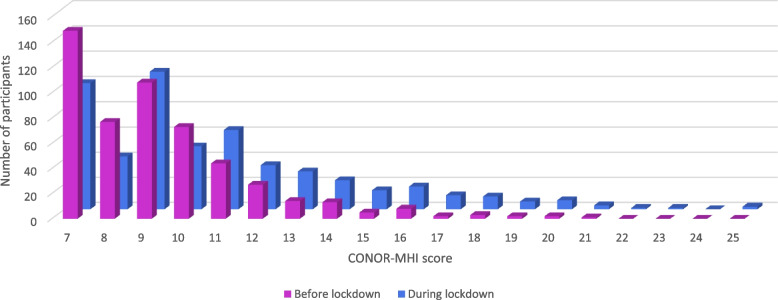
Mental health before and during the lockdown. Distribution of participants’ CONOR-MHI total score (7-28), where higher scores indicate wors mental health. No participants had more than 25 points

In total, 44.6% of the participants reported being less active or much less active during the lockdown compared to before the lockdown, 46.5% reported being as active as before lockdown, and 8.3% reported being more active than before the lockdown. The Wilcoxon signed-rank test indicated lower levels of PA (Z = -4.918, *p* < 0.001) and worse general and mental health (Z = -6.910, *p* < 0.001; Z = -12.114, *p* < 0.001) during the lockdown than before (see Table 2). The same analysis for the HUNT 1 PA-Q subscales indicated significant lower frequency (Z = -3.737, *p* < 0.001), intensity (Z = -6.540, *p* < 0.001) and duration (Z = -7.909, *p* < 0.001) of PA during lockdown. Little to no change is seen in median values, but Figs. 1, 2 and 3 illustrates a significant shift of the participants’ answers, from positive to negative.

**Table 2:** Changes in PA and health from before to during the lockdown

Variables^a^	Negative Ranks			Positive ranks			Test statistics			Median before lockdown	Median during lockdown
	*n*	Mean rank	Sum of ranks	*n*	Mean rank	Sum of ranks	Ties	Z	*p*		
(PA before lockdown) - (PA during lockdown)	131	98.66	12,925	60	90.18	5411	285	-4.918	< 0.001	5.5	5.5
(General health before lockdown) - (General health during lockdown)	14	45.86	642	83	49.53	4111	464	-6.910	< 0.001	3	3
(Mental health before lockdown) - (Mental health during lockdown)	49	108.33	5308	264	166.03	43,833	199	-12.114	< 0.001	9	10

^a^A decrease in PA score and an increase in health scores are considered a negative change

### Changes in health based on PA levels during lockdown

Adjusted for gender and type of activity, the odds of having worse general and mental health during the lockdown were significantly higher for those who did not maintain their activity level, with the odds ratio being 9.36 (95% CI = 4.71–18.58, *p* < 0.001) for general health and 2.41 (95% CI = 1.52–3.83, *p* < 0.001) for mental health. There were no significant higher or lower odds for change in health related to gender or type of exercise (Tables 3, 4 and 5).

**Table 3:** General health based on PA levels during lockdown

Variable	Exp(B)	95% Confidence interval		Sig. (*p*)
		**Lower**	**Upper**	
**PA lockdown vs March** Less active As active or more (ref.)	9.359	4.713	18.584	< 0.001
**Gender** Female Male (ref.)	1.469	0.718	3.007	0.292
**Type of exercise** Organized offer ex Non-organized ex. (ref.)	1.243	0.716	3.366	0.265

**Table 4:** Mental health based on PA levels during lockdown

Variable	Exp(B)	95% Confidence interval		Sig. (*p*)
		**Lower**	**Upper**	
**Activity lockdown vs March** Less active As active or more (ref.)	2.412	1.520	3.827	< 0.001
**Gender** Female Male (ref.)	1.430	0.938	2.181	0.097
**Type of exercise** Organized offer ex Non-organized ex. (ref.)	1.357	0.886	2.077	0.160

**Table 5:** Changes in PA based on type of activity

Variable	Exp(B)	95% Confidence interval		Sig. (*p*)
		**Lower**	**Upper**	
**Type of exercise** Ex. through organized offers Non-organized ex. (ref.)	3.046	1.871	4.958	< 0.001
**Gender** Female Male (ref.)	1.035	0.655	1.635	0.882
**Health** Good Somewhat good Bad Excellent/very good (ref.)	1.284 1.520 2.053	0.810 0.774 0.514	2.036 2.947 8.200	0.288 0.227 0.309

### Changes in PA levels based on type of activity

Adjusted for gender and health status, the odds ratio of being less active during lockdown was significantly higher for those who attended organized offers of exercise before lockdown. Compared with those who did not exercise in organized offers before lockdown, the odds ratio for being less active as a pre-lockdown organized offer exerciser was 3.05 (95% CI = 1.87–4.896, *p* < 0.001).

There were no significant higher or lower odds for changes in PA related to gender or health.

## Discussion

We investigated how physical activity, general health, and mental health in community-dwelling older adults were affected by the COVID-19 pandemic in Norway, and we found a significant decrease in physical activity, general health, and mental health during the period of restrictions compared to before the lockdown. Furthermore, we found that those who were less active during lockdown had an odds ratio of 9.36 of having worse general health and an odds ratio of 2.41 of having worse mental health compared to those who maintained their activity levels. Lastly, we found that “organized offer exercisers” had an odds ratio of 3.21 of being less active during lockdown compared to “non-organized exercisers”.

Our results showing a reduction in activity level and in general and mental health after the onset of COVID-19 restrictions are closely aligned with other recent studies and reports [22–24]. A narrative review assessing the impact of social isolation on health in older people found that six of the eight studies reported an increased level of psychological stress, which included higher levels of anxiety, depression, and loneliness [22]. Furthermore, Yamada and colleagues found that non-frail older adults (mean age: 73.5 years) had decreased their total PA time by 40% compared to the pre-pandemic period [25]. Additionally, the Active Lives Adult Survey found a 7.3% drop in activity levels from March 2020 for older adults aged 55–74 years and 6.6% drop for those aged 75 years and up [23]. The same survey found that the proportion of the overall population classified as ‘inactive’ increased by 7.4% during the pandemic.

The reductions in PA and general health in our study were significant but nonetheless quite small, and they did not change all median values. We have not found articles reporting data on minimal clinically important differences with the measurements we used, so we cannot say if our findings are clinically meaningful. Still, the changes for all three variables are in a negative direction, and they might have an additive effect on each other. The lockdown was introduced in March 2020, when it was still cold and snowy in many parts of Norway, while the during-lockdown measures were assessed in the summer, when activity is normally higher [6, 26]. Because of this, the small change we found might be important.

We included home-dwelling older adults with a wide range in age and a relatively good gender balance (40/60 men/women). Our participants were better educated than participants from Statistics Norway, with almost 60% of the former having a university degree compared to only 24.6% of the total population aged ≥ 67 years, according to Statistics Norway [27]. Participants were more active than participants in the HUNT study [28], with 84% of our participants reporting being physically active more than once per week compared to 75% in the HUNT study. The level of PA increases with higher levels of education [6]. Visser and colleagues found that having functional limitations were associated with a negative impact of the COVID-19 pandemic on PA and exercise [29]. Still, with our highly educated, physically active, and well-functioning sample, we found a decrease in both PA and health. It could be argued that older adults with more advanced age and/or lower physical function than the participants in the current study might have been even more negatively affected by COVID-19 restrictions.

We found that maintaining one’s activity level during lockdown was associated with better general and mental health. Carriedo [30] and colleagues found similar results, where older adults who met the global recommendations for PA during quarantine in Spain had better mental health than those who did not engage in PA; these results are supported by reviews showing a positive association between PA and health [31, 32].

Those participating in organized offers of exercise had higher odds of becoming less active during the pandemic compared to those who did not engage in organized offers of exercise. This illustrates that participants attending organized exercise may get dependent on the exercise offer to maintain activity levels. This finding can be supported by Henderson and colleagues, who found that participants did not continue their activity level after an exercise intervention stopped [33]. A low threshold for participation and a social setting, provided by many organized exercise offers, can motivate and stimulate some to higher levels of PA. Thus, during special times (like the COVID-19 pandemic lockdown), keeping PA and exercise offers open should be a priority. If this is not possible it should be of public interest to come up with alternatives such as offers adapted to older people that can be done at home. Examples include exercise offers delivered through TV-programs, digital solutions (either real-time or as a video bank), or leaflets that can be sent by mail or printed. In Norway, some gyms transferred some of their group exercise classes to digital platforms, but few of these offers were adapted to older adults, both regarding user friendliness and content of the exercise. Although a large proportion of older adults in Norway use screen-based media or smartphones [34, 35], a survey shows that this proportion decreases among those over 80 years and that only 8% of people over the age of 70 years in Norway had been in contact with healthcare personnel using telephones or screens during the COVID-19 pandemic [34]. Public Health England has estimated that 100,000 more older people will have at least one fall per year as a result of reduced PA during the pandemic, with annual health and social care system costs of £211 million [36]. The same report estimates that by increasing strength and balance activity by 10% in all older adults compared to the levels observed during the pandemic, could reduce the number of falls by more than 9000 and costs of £16 million. As the proportion of older adults who falls each year is similar between Norway and England [37–39] it is to be believed that some of the costs needed to develop user-friendly digital or TV-based exercise for older adults would be saved on costs related to falls. Hargreaves and colleagues found that vigorous and moderate intensity PA were significantly lower both during- and post lockdown compared to pre lockdown in those who had been highly active pre-lockdown [40]. This demonstrates difficulties in motivating oneself to get back to one’s usual levels of PA after a period of less PA. It could be argued that the older adults who experienced a decrease in PA and exercise during the pandemic might stay less active, even if society goes back to its normal state. Even if they eventually get back to their pre-pandemic levels of PA, it is not clear how long this would take. Worse general and mental health could also contribute to less motivation to be active [41]. In our study, maintaining one’s level of PA had a protective effect on mental health, and it is possible that a negative spiral of less PA leads to reduced mental health, which leads to even less PA.

### Strengths and limitations

The strength of this study is its design, which made it possible to assess PA, general health, and mental health—which are important aspects of function in old age—during a period of lockdown in society, thus assessing change in these variables in a natural experiment. Furthermore, we used PA and health items that have also been used in other large studies and are thus able to compare our participants’ characteristics with those of other studies.

We cannot rule out a self-reporting bias, as all the variables are based on answers from a questionnaire. Many of the questions were also about retrospective experiences, which could have caused a recall bias.

Because our sample consists of relatively healthy, well-educated older persons, the results cannot be generalized to the overall population of older adults.

The data were collected in the summer, 3–5 months after the lockdown began; some restrictions had already been lifted at that point. Our results are based on the answers we obtained at this specific time period. We do not know whether we would have obtained the same answers immediately after the lockdown began, or at a longer time after the onset of the restrictions.

## Conclusions

We included a relatively highly educated and active group of older participants in this study, but COVID-19 restrictions still negatively affected both their levels of activity and their general and mental health. Maintaining activity levels throughout the lockdown seems to be associated with better general and mental health during the lockdown. Those attending group exercise before the lockdown were more negatively affected by the restrictions than those who exercised individually. The long-term consequences of the pandemic on health and function in older adults have not yet been established, but our and other results show short-term decreases in activity level and health, indicating that preventive actions and increased focus on measures to help older adults maintain an active lifestyle are needed.

Future research should include more vulnerable and frail older adults. 

## Data Availability

The dataset generated and/or analyzed during the current study are not publicly available until after five years due to ethical considerations. The dataset can be made available from the corresponding author on reasonable request, however, this is not applied for in the ethical application and must so be done before data sharing.
